# Effects of middle-aged and elderly people’s self-efficacy on health promotion behaviors: Mediating effects of sports participation

**DOI:** 10.3389/fpsyg.2022.889063

**Published:** 2023-01-04

**Authors:** Nan Chen, Jia Zhang, Zhiyong Wang

**Affiliations:** ^1^School of Physical Education, Anyang Institute of Technology, Anyang, China; ^2^School of Physical Education, Chongqing University, Chongqing, China; ^3^Pain Department, Anyang City Third People’s Hospital, Anyang, China

**Keywords:** self-efficacy, sports participation, health promotion behaviors, middle-aged, mediating effect

## Abstract

**Objective:**

This study explores the relationship between self-efficacy, sports participation, and health promotion behavior for middle-aged and elderly people. Therefore, it provides a theoretical reference for improving the quality of life for middle-aged and elderly adults and promoting a healthy lifestyle for the elderly.

**Methods:**

A total of 591 (men: 36.2%; women: 63.8%; age: above 50 years) middle-aged and elderly adults from five cities of Henan Province were selected as the research objects by convenient sampling. The self-efficacy, sports participation, and health promotion behavior scales were used for the questionnaire survey. Amos24.0 was used to test the structural equation model, intermediary function test, and bootstrap analysis. Results: The self-efficacy of middle-aged and elderly people positively impacted health promotion behavior. The path coefficient was 0.439. Sports participation played a partial intermediary role between self-efficacy and health promotion behavior (χ*^2^/df* = 1.785, root mean square error of approximation = 0.036, root mean square residual = 0.021, goodness-of-fit index = 0.967, comparative fit index = 0.976, Tucker–Lewis Index = 0.971) The proportion of intermediary effect was 26.34% (0.100, 0.225).

**Conclusion:**

(1) Self-efficacy can significantly and positively affect health promotion behavior for middle-aged and elderly people; (2) sports participation plays a partial intermediary role between self-efficacy and health promotion behavior. From this point of view, we can enhance the self-efficacy of middle-aged and elderly people and improve their healthy life behavior by advancing sports participation. Thus, it provides theoretical support and practical guidance for promoting national health.

## Introduction

According to the demographic data released by the National Bureau of Statistics of China in 2020, people aged over 45 years account for about 42.6%, while those aged over 65 years account for about 13.5% of the total population (National Bureau of Statistics [NBS], 2021). By the end of 2050, it is predicted that over 480 million people in China will be over 65 years old (Yang, 2022). Regarding middle-aged people, the gradual decline in physiological function makes them more prone to cardiovascular and cerebrovascular diseases and various degenerative dysfunction diseases, and the heavy responsibilities from both family and society bring about severe mental conflicts, which makes their health conditions a tricky problem in social development (Zhang et al., 2011). In recent years, diseases caused by unhealthy lifestyles, such as cardiovascular diseases and malignant tumors, have become the leading factor in the death of humans (Feng, 2013). Studies have indicated that 60% of people’s health and life span depend on their behaviors and lifestyles (Jiang and Wu, 2009). Consequently, we could effectively prevent chronic diseases and improve quality of life by improving lifestyles and health promotion behaviors (Chassaing et al., 2014; Burcelin, 2016; Jeong and Jang, 2016).

Health promotion behaviors refer to the health management activities for a healthier life that inspires people’s potentiality of health and advances their physical and mental health (Hee, 2021). Baker (2007) developed six parts of daily health promotion behaviors, namely, self-actualization, health responsibility, physical exercise, nutritional diet, interpersonal support, and stress management. Health promotion behaviors might be influenced by care from family members, personal attitudes toward health, and physical exercise (Chu and Sang, 2019). According to Lee and Oh (2020), confidence in performing health behaviors, i.e., self-efficacy, plays a crucial role in influencing the decision-making of health promotion behaviors. Hence, people with a high level of self-efficacy would prefer healthy life behaviors.

Observed from the existing relative literature, self-efficacy might affect health promotion behaviors in four aspects: self-efficacy is an essential predictor of tobacco and alcohol withdrawal behavior (Keller et al., 2016); it has a positive effect on the behavior of chronic patients [e.g., control of blood sugar (Osborn et al., 2010), control of rheumatoid arthritis pain (Martinez et al., 2020)]; it can improve patient compliance with medication (Van Herpen-Meeuwissen et al., 2022); and it can promote adherence to physical exercise (Zhu et al., 2018). For patients with diabetes, self-efficacy is independent of exercise and diet (King et al., 2010). Studies on self-efficacy in health promotion have gradually become the research hotspot in the past few years with no prediction mechanism involved. There might be significant amounts of intervening variables in the influence of self-efficacy on the health promotion behaviors of middle-aged and elderly people. As an essential indicator, sports participation positively affects health promotion behaviors. Wu and Liu (2013) held that self-efficacy is an essential predictor of sports participation. Lee and Oh (2020) found the full intermediate effect of sports participation when studying the effect of adolescent health literacy on health promotion behaviors.

In conclusion, studies have indicated that there is a positive correlation between self-efficacy and sports participation, and health promotion behaviors, but its internal mechanism remains unclear. The structural equation model (SEM) might be applied herein to analyze the intermediary role of sports participation in self-efficacy and health promotion behaviors to identify the internal mechanism of the influence of self-efficacy on health promotion behaviors based on the existing research results and practices and provide theoretical support and empirical data for comparison for the health management behaviors and disease prevention of middle-aged and elderly people to keep fit.

## Literature review and research hypotheses

### Influence of self-efficacy on health promotion behaviors

As proposed by the psychologist Bandura, self-efficacy refers to one’s confidence in his/her abilities to achieve behavioral objectives in a particular field, which will directly affect one’s behavioral motivation. Bandura (1997) suggested that self-efficacy plays an essential role in initiating and maintaining human behavior. First, people’s lives are full of decisions on how to act and how long the action will last. Their judgment of self-efficacy determines their choice of social environment and behavior, i.e., individuals tend to choose what they feel they can handle but avert situations and tasks beyond their abilities (Erdem and Demirel, 2007). Second, self-efficacy judgment determines how hard and long a person will persist when he/she encounters an obstacle or unpleasant experience. If difficulties keep emerging, those who doubt their abilities will slacken off or even give up, whereas those with stronger self-efficacy will strive to tackle the daunting challenge (Panadero et al., 2017). These studies have demonstrated that self-efficacy is an essential factor in the completion of highly challenging tasks. A person with a high sense of self-efficacy tends to have more confidence in his/her abilities and more self-control in daily life.

The World Health Organization (WHO) defines health promotion as the process during which people can better control and improve their health (Lawrence and John, 1988). In 2009, the *American Journal of Health Promotion* identified health promotion as an art and science that helps people discover the synergistic effects between their core passion and optimal health, strengthens their motivation for optimum health and changes their lifestyles to approach the highest health level. Pender (1996) proposed a perspicuous and comprehensive definition based on the health promotion model, i.e., health-promoting behaviors are individuals’ long-term multidimensional activities to exhibit their optimal health conditions and realize self-fulfillment. Hence, the health-promoting model can improve people’s quality of life (Kumar and Preetha, 2012).

Meanwhile, it is found that self-efficacy is a cognitive factor that affects people’s health-promoting lifestyles, willingness, and motivation to perform healthy behaviors (Zhao, 2011). Luan et al. (2020) found that elderly people with a high level of self-efficacy are more confident in maintaining health and developing healthy living habits in their research on the elderly in urban and rural areas. The study of Jiang (2015) demonstrated that self-efficacy plays a positive role in sports participation, self-actualization, and pressure handling of patients with chronic cardiac failure. Based on the above analysis, this study puts forward the following hypothesis:

Hypothesis 1: The self-efficacy of middle-aged and elderly people exerts a significant positive impact on their health-promoting behaviors.

### Self-efficacy and sports participation

The initial definition of sports participation primarily reflects the state of an individual’s external behavior in group activities manifested by the presence or absence of the body. With the development of the psychological perceptions of participants in sports participation, researchers have expanded the concept of sports participation to participants’ cognitive and emotional states in group activities (Liu and Yu, 2005). The concept of participation emphasizes both external quantitative manifestation and internal qualitative requirements. In education, numerous scholars have proposed a new viewpoint about the definition and measurement dimensions of student participation, i.e., the degree of participation serves as an essential indicator of participation behavior. Westbrook and Oliver (1991) suggested that behavioral change is a three-stage process, i.e., “cognition-emotion-behavior.” By summarizing previous research, Fredricks et al. (2004) classified learning participation into behavioral, emotional, and cognitive participation, each of which is not independent. Meanwhile, Chinese researchers have localized, generalized, and summarized the related theories. The research by Kong (2005) indicated that the definition of student participation primarily involves three dimensions, i.e., behavioral, cognitive, and emotional. The three-dimensional definition of student participation is comparatively recognized by academia and continues to be developed based on this. Given the above research on sports participation, the present study defines sports participation as the engagement level of middle-aged and elderly people in sports behavior, sports emotion, and sports cognition in sports activities.

Studies have indicated that self-efficacy and physical training behaviors are mutually beneficial (Zhang and Zha, 2017), i.e., self-efficacy maintains exercise intentions and behaviors, and regular exercise, in turn, helps improve self-efficacy. As related studies indicate, self-efficacy has influenced sports participation in the following aspects. First, self-efficacy affects people’s behavioral intentions and attitudes toward sports participation (Wu and Li, 2016). Second, it influences the regularity and persistence of people’s sports participation. The higher the level of self-efficacy, the more regular the exercise behaviors and the firmer the core belief that drives long-term physical exercise behaviors (Sheng et al., 2016; Dong et al., 2018). Notably, self-efficacy plays a remarkable role in people’s sports participation. Based on the above analysis, this study puts forward the following hypothesis:

Hypothesis 2: Self-efficacy imposes a significant positive impact on sports participation.

### Sport participation and health-promoting behaviors

Regular exercise exerts a substantial impact on health promotion. The study by Eime et al. (2020) demonstrated that those in good health had regular exercise habits, whereas those with diseases did less exercise. Sports activities are planned and organized repetitive physical activities that aim at improving or maintaining people’s physical fitness (Anang et al., 2019). Among the factors that affect the prevention of adult diseases, exercise, nutrition, and rest are essential. A previous study has demonstrated that regular sports participation will affect the perceived health status and the persistence of health-promoting behaviors (Morgan et al., 2016). Physical exercise is conducive to enhancing people’s health awareness, forming a healthy lifestyle (Meng, 2013), and promoting the psychological and emotional regulation ability of sports participants (Wang et al., 2016; Brière et al., 2018). Moreover, the intervention of physical exercise exerts a significant impact on the health promotion behaviors of patients during treatment and recovery from chronic diseases (Dashtidehkordi et al., 2019). Based on the above analysis, this study puts forward the following hypothesis:

Hypothesis 3: Sports participation imposes a significant positive impact on health-promoting behaviors.

### The intermediary role of sports participation in the relationship between self-efficacy and health-promoting behaviors

As observed from previous studies, sports participation is an intermediary between individual cognitive ability and actionability. People with higher self-efficacy are likely to exhibit a higher level of sports participation. Lee et al. (2020) found that sports participation imposes an intermediary impact on the relationship between health awareness and health-promoting behaviors. Therefore, the self-efficacy of middle-aged and elderly people might affect their health-promoting behaviors through the mediating effect of sports participation. Based on the above analysis, this study puts forward the following hypothesis:

Hypothesis 4: The sports participation of middle-aged and elderly people plays an intermediary role between their self-efficacy and health-promoting behaviors.

## Materials and methods

### Study design

Middle-aged and elderly people from five cities in the east, west, south, north, and middle of Henan Province (Shangqiu city, Jiaozuo city, Xinyang city, Anyang city, and Zhengzhou city) were selected as the research samples. The selected objects cover a wide range, and the sampling points are relatively scattered, which can better represent the overall situation of Henan Province. Inclusion criteria were (1) local permanent resident population ≥ 45 years old; (2) informed consent and voluntary participation in the study; and (3) have apparent language expression ability and reading ability and have no obstacle to communicating with investigators. Exclusion criteria were (1) serious language and hearing disorders, (2) serious mental illness patients, and (3) people with dementia who cannot precisely answer questions.

From September to November 2021, through the convenient sampling method, investigators conducted a questionnaire survey on middle-aged and elderly people who were engaged in leisure activities in the elderly activity centers, parks, and city squares of these five cities. In the research sample, the personal characteristics were extracted from four parts, namely, gender, age, education level, and economic status. Before the survey, they issued an “Informed Consent Form for the Survey.” They observed that the informed and consent respondents returned the questionnaire immediately after filling it in the questionnaire.

According to the sampling standard developed by Ghiselli et al. (1982), if a survey is conducted on a scale, the sample size should be at least 10 times the total number of questions in the scale. The three scales applied in the present study consist of 59 questions. Hence, 590 valid data should be collected. There are 130 questionnaires distributed in each city and 650 in total. A total of 591 valid questionnaires, excluding 59 incomplete and dishonest ones, were retained. The effective rate is 90.9%.

The descriptive statistics of demographic information are shown in Table 1. There are 214 male respondents, accounting for 36.2%. There are 377 women, accounting for 63.8%. According to the WHO age classification standard, middle-aged people are defined as those over 45 years old, and elderly people are defined as those over 65 years old. All the samples in this study are over 50 years old. *Age grouping*: According to Zhang (2017), in his research, the standard that middle-aged and elderly people are divided into groups every 5 years old is adopted. The leading group is the educational level of high school/technical secondary school graduates. Tan et al. (2020) indicates that China has a large land, and the economic development between the east and the west needs to be balanced. The results of the economic status survey are as follows: 24 people are tough, accounting for 4.1%, and 12 people are complex, accounting for 2.0%, 482 people report general at most, accounting for 81.6%, and 73 people report wealthy, accounting for 12.4%.

**TABLE 1:** The basic situation of the respondents.

Variables	Category	Frequency	Proportion (%)
Gender	Men	214	36.2
	Women	377	63.8
Age	50–54 years old	110	18.6
	55–59 years old	122	20.6
	60–64 years old	231	39.1
	65–69 years old	76	12.9
	70–74 years old	16	2.7
	75–79 years old	26	4.4
	Over 80 years old	10	1.7
Education level	Primary school	26	4.4
	Junior middle school	99	16.8
	High school	250	42.3
	college	151	25.5
	Bachelor degree and above	65	11
Economic conditions	Very poor	24	4.1
	Poor	12	2.0
	General	482	81.6
	Rich	73	12.4

### Research tools

The Self-Efficacy Scale was revised by Wang (2002). There are 10 questions in total, which features few questions and simple operation, referring to a one-dimensional scale adopting the 5-point scoring of the Likert scale with only the total scale score being calculated in the evaluation and a higher score representing a higher level of self-efficacy.

The Sports Participation Scale is based on the sports participation classification model developed by Snyder and Spritzer and refers to the sports participation scale formulated by Zou (2017). There are 13 questions which interpret the sports participation level from three dimensions–cognitive sports participation, emotional sports participation, and behavioral sports participation and adopt the 5-point scoring of Likert with higher scores representing a higher level of sports participation.

The Health-Promoting Lifestyle Profile-II, revised by Pullen et al. (2001) following HPLP, covers 52 items and six dimensions, namely, health responsibility, nutrition, physical activities, interpersonal relationships, stress management, and self-actualization, and adopts the 5-point scoring of Likert scale with higher scores representing better health-promoting behaviors, which was translated into Chinese by Shi and Li (2003). According to the study of Kim (2013), exercise and nutrition both belong to personal management behaviors; referring to the research of Kwon and Kang (2018), this study classifies exercise and nutrition as one factor.

Cronbach’s α coefficient of the Self-Efficacy Scale is 0.885, more significant than the benchmark of 0.7, and the composite reliability (CR) is 0.899, more significant than the benchmark of 0.6, implying good internal consistency. The average variance extracted (AVE) value is 0.531, which is more significant than the benchmark of 0.5. The above values conform to the research standards that Bagozzi and Yi (2011) and Joseph et al. (2012) proposed. According to the viewpoint proposed by Hu and Bentler (1999), the value of χ*^2^/df* should be less than 3 in a fit index of a good model, the value of the goodness-of-fit index (GFI), comparative fit index (CFI), Tucker–Lewis index (TLI) should be greater than 0.9, and the value of root mean square error of approximation (RMSEA) and root mean square residual (RMR) should be less than the standard of 0.08. The analysis results of confirmatory factor are χ*^2^/df* = 2.042, GFI = 0.987, CFI = 0.992, TLI = 0.998, RMSEA = 0.042, and RMR = 0.018.

As indicated by the confirmatory factor analysis results in Tables 2, 3, the scale has a good fitting, and the grading results have high reliability and validity. The Cronbach’s α coefficient of the sports participation scale is 0.832, and the three dimensions are 0.826, 0.779, and 0.846, respectively, which are greater than the benchmark of 0.7, implying good internal consistency. CRs are 0.842, 0.753, and 0.846, which are greater than the benchmark of 0.6, implying good internal consistency. The values of AVE are 0.641, 0.580, and 0.568. The confirmatory factor analysis results are χ*^2^/df* = 1.994, GFI = 0.975, CFI = 0.983, TLI = 0.977, RMSEA = 0.041, and RMR = 0.023.

**TABLE 2:** Fitting indexes of confirmatory factor analysis.

Variables	χ*^2^/df*	GFI	CFI	TLI	RMR	RMSEA
Self-efficacy	2.042	0.987	0.992	0.998	0.042	0.018
Sports participation	1.994	0.975	0.983	0.977	0.041	0.023
Health promotion	1.650	0.940	0.974	0.970	0.033	0.025

**TABLE 3:** Reliability and convergent validity of the study constructs.

Construct	Item	Standard loading	CR	Cronbach’s α	AVE
Self-efficacy (SE)	SE1	0.619	0.899	0.885	0.531
	SE2	0.749			
	SE3	0.738			
	SE4	0.754			
	SE5	0.768			
	SE6	0.696			
	SE10	0.748			
Cognitive sports participation (CSP)	CSP1	0.790	0.842	0.826	0.641
	CSP2	0.775			
	CSP4	0.784			
Emotional sports participation (ESP)	ESP6	0.731	0.753	0.779	0.580
	ESP7	0.703			
	ESP8	0.773			
Behavioral sports participation (BSP)	BSP9	0.773	0.867	0.846	0.568
	BSP10	0.696			
	BSP11	0.729			
	BSP12	0.683			
	BSP13	0.739			
Self-actualization (SA)	SA1	0.794	0.848	0.786	0.529
	SA2	0.823			
	SA3	0.654			
	SA4	0.703			
	SA5	0.658			
	SA6	0.694			
Health responsibility (HR)	HR7	0.788	0.910	0.885	0.650
	HR8	0.724			
	HR9	0.658			
	HR10	0.850			
	HR11	0.829			
	HR12	0.673			
Sports nutrition (SN)	SN14	0.603	0.911	0.756	0.632
	SN15	0.717			
	SN16	0.735			
	SN17	0.838			
	SN21	0.687			
	SN24	0.801			
Interpersonal relationship (IR)	IR25	0.842	0.912	0.861	0.675
	IR26	0.803			
	IR27	0.745			
	IR28	0.740			
	IR30	0.685			
Stress management (SM)	SM32	0.758	0.883	0.792	0.650
	SM33	0.827			
	SM34	0.725			
	SM36	0.693			

The confirmatory factor analysis in Tables 2, 3 indicates that the scale fits well. The Cronbach’s α coefficient was 0.9. Some items in this scale that might cause difficulties in language comprehension due to cultural differences or non-conformance to the focus of this study were deleted, and 36 items were applied to measure health-promoting lifestyles. There are four dimensions for the scale in this study, with Cronbach’s α coefficient being 0.813 and α for dimensions being 0.861 (interpersonal relationship), 0.786 (self-actualization), 0.792 (stress management), 0.885 (health responsibility), and 0.756 (sports nutrition), which are all greater than the benchmark of 0.7. CRs are 0.848, 0.910, 0.911, 0.912, and 0.883, respectively, which are all greater than 0.7, implying good internal consistency. The AVE values are 0.529, 0.650, 0.632, 0.675, and 0.650, which are all greater than the benchmark of 0.5. The confirmatory factor analysis results are χ*^2^/df* = 1.650, GFI = 0.940, CFI = 0.974, TLI = 0.970, RMSEA = 0.033, and RMR = 0.025, As indicated by the confirmatory factor analysis of the scale in Tables 2, 3, all fitting indexes could meet the requirements.

### Data processing

The data into SPSS 24.0 and AMOS 24.0 analysis software were imported, the invalid questionnaires were removed, the valid data were processed, and the score was calculated. The measurement tool’s reliability, content validity, and construct validity were tested through reliability analysis, exploratory factor analysis, and confirmatory factor analysis. The standard distribution and parameter tests of relevant variables were conducted using descriptive statistics. After the standardized data processing, the direct influence of self-efficacy and sports participation on health promotion behaviors was investigated through correlation analysis, regression analysis, and other methods. The bootstrap method was used to repeatedly sample 5,000 times to obtain a 95% confidence interval of the coefficient and the mediating effect, and the indirect influence of self-efficacy on the health promotion behavior of middle-aged and elderly people was analyzed, i.e., the mediating effect of sports participation was investigated.

## Results

### Common method bias test

To test the possible standard method bias, Harman single-factor test was conducted on the data (Podsakoff et al., 2003). The principal component analysis results for rotation indicated that there were nine factors with an eigenvalue greater than 1, of which the first one had a variance of 27.70%, lower than the critical value of 40%, implying that there was no apparent standard method bias in this study. The hypotheses in this study might be tested following the latest test procedures for intermediate effect proposed by Wen and Ye (2014).

### Correlation analysis of self-efficacy, sports participation, and health promotion behaviors of middle-aged and elderly people

The results of Pearson product–moment correlation analysis applied herein indicate that the average score of self-efficacy is 3.57 ± 0.717, and scores for three dimensions of sports participation, including cognitive participation, emotional participation, and behavioral participation, are 3.62 ± 0.792, 3.62 ± 0.771, and 3.65 ± 0.721, respectively. Those for the five dimensions of health promotion behaviors, including self-actualization, health responsibility, sports nutrition, interpersonal relationships, and stress management, are 3.68 ± 0.697, 3.79 ± 0.672, 3.81 ± 0.643, 3.81 ± 0.665, and 3.82 ± 0.673, respectively. Table 4 shows a positive correlation among all dimensions with correlation values between 0.161 and 0.468, implying that self-efficacy, sports participation, and health promotion behaviors of middle-aged and elderly people are highly related.

**TABLE 4:** Correlation results.

Variables	M ± SD	SE	CSP	ESP	BSP	SA	HR	SN	IR	SM
SE	3.57 ± 0.717	1								
CSP	3.62 ± 0.792	0.305**	1							
ESP	3.62 ± 0.771	0.400**	0.310**	1						
BSP	3.65 ± 0.721	0.246**	0.302**	0.341**	1					
SA	3.68 ± 0.697	0.368**	0.398**	0.399**	0.376**	1				
HR	3.79 ± 0.672	0.457**	0.347**	0.468**	0.427**	0.478**	1			
SN	3.81 ± 0.643	0.437**	0.397**	0.408**	0.422**	0.317**	0.320**	1		
IR	3.81 ± 0.665	0.406**	0.340**	0.379**	0.352**	0.324**	0.315**	0.361**	1	
SM	3.82 ± 0.673	0.353**	0.259**	0.348**	0.264**	0.183**	0.225**	0.316**	0.161**	1

**p < 0.01.

SE, self-efficacy; CSP, cognitive sports participation; ESP, emotional sports participation; BSP, behavioral sports participation; SA, self-actualization; HR, health responsibility; SN, sports nutrition; IR, interpersonal relationship; SM, stress management.

### SEM model building of self-efficacy and health promotion behaviors of middle-aged and elderly people

**FIGURE 1:**
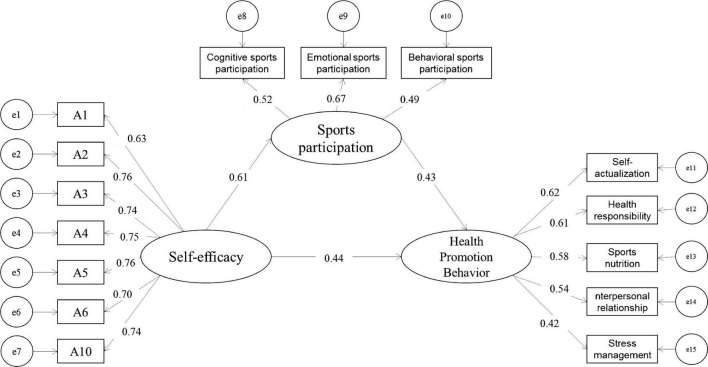
Path analysis.

To verify the hypotheses, self-efficacy was applied as an independent variable, health promotion behaviors as a dependent variable, and sports participation as an intermediary variable in the SEM model in this study to build an equation model *via* Amos24.0. Figure 1 shows the path coefficient between the estimated parameters of the SEM and variables. The model’s goodness of fit was χ^2^/*df* = 1.785, RMSEA = 0.036, RMR = 0.021, GFI = 0.967, CFI = 0.976, and TLI = 0.971, respectively, implying that the model has a good fitting.

The percentile bootstrap method for deviation correction was applied herein to test the intermediate effect. The hypotheses were tested by calculating a 95% confidence interval after repeated resampling 5,000 times. According to Table 5 and Figure 1, the coefficient of self-efficacy to health promotion behaviors in the model is 0.439 (*p* < 0.001, 95% confidence interval [0.179, 0.358], 0 excluded), hence, Hypothesis 1 is verified. The path coefficient of self-efficacy to sports participation is 0.609 (*p* < 0.001, 95% confidence interval [0.277, 0.441], 0 excluded); the path coefficient of sports participation to health promotion behaviors is 0.430 (*p* < 0.001, 95% confidence interval [0.286, 0.640], 0 excluded); and the intermediate effect value of self-efficacy on health promotion behaviors through sports participation is 0.157 (*p* < 0.001, 95% confidence interval [0.100, 0.225], 0 excluded). Given the above, Hypotheses 2–4 are supported, i.e., sports participation plays an intermediary role in the effect of self-efficacy on health promotion behaviors, which accounts for 26.34% in the total effect, i.e., 26.34% of the total effect of self-efficacy on health promotion behaviors are realized through sports participation, implying that sports participation plays a partial intermediary role in the effect of self-efficacy on health promotion behaviors.

**TABLE 5:** Hypotheses model path and effect decomposition.

Classes	Path	B	S.E.	C.R.	95% confidence interval	Effect ratio
Direct effect	Self-efficacy → Health promotion behaviors	0.439***	0.043	6.059	0.179, 0.358	73.66%
	Self-efficacy → Sports participation	0.609***	0.043	8.316	0.277, 0.441	
	Sports participation → Health promotion behaviors	0.430***	0.091	4.873	0.286, 0.640	
Intermediary effect	Self-efficacy → Sports participation → Health promotion behaviors	0.157***	0.031		0.100, 0.225	26.34%

***p < 0.001.

## Discussion

### Effect of self-efficacy on health promotion behaviors of middle-aged and elderly people

According to the research results, self-efficacy had a significant positive effect on health promotion behaviors for middle-aged and elderly people, i.e., the higher the self-efficacy of middle-aged and elderly people, the higher level of their health promotion behaviors, which verifies Hypothesis 1 and is consistent with the conclusion of Fisher and Kridli (2014) and Luan et al. (2020), who found in their research on the elderly with osteoporosis in the community that the elderly with a high level of self-efficacy have a more balanced diet and more regular sports performance (Li and Chen, 2005). As indicated by the survey of 4,040 villagers aged 40–69 years in Wanxi, Anhui province, by Liang et al. (2016), self-efficacy could predict health behaviors, i.e., villagers with higher self-efficacy are more confident in keeping healthy and more persistent in healthy behaviors. Studies on the effect of self-efficacy on healthy lifestyles have indicated that self-efficacy is the essential influencing factor in health promotion lifestyles and health self-management (Feng et al., 2014; Zheng et al., 2020). Self-efficacy refers to people’s confidence in judging whether they can complete a specific task. Hence, it is an important influencing factor on personal behaviors, i.e., a higher level of self-efficacy leads to higher self-esteem and confidence, rational cognitive behavior and self-assessment, and increased success rate (Zhao et al., 2014). Therefore, people with higher self-efficacy are more likely to overcome difficulties in daily life, control their behaviors, and develop healthy life behaviors.

### Mediating effect of sports participation between self-efficacy and health promotion behaviors of middle-aged and elderly people

Structural equation model analysis has indicated that self-efficacy directly affects health promotion behaviors and indirectly affects them through sports participation. Meanwhile, bootstrap results show that the intermediate effect of sports participation in the effect of self-efficacy on health promotion behaviors accounts for 26.34% in a 95% confidence interval with *p* < 0.001, which verifies its significant indirect effect, hence, Hypotheses 2–4 are true. Yang (2019) and Arribas et al. (2020) found that self-efficacy has a significant positive effect on sports participation, and Wu and Liu (2016) held that self-efficacy is an essential predictor of people’s attitudes toward exercise, which are consistent with the results of this study. Self-efficacy is generally regarded as a subjective judgment of an individual about whether he or she could successfully engage in a particular behavior and about his or her confidence in getting things done. Therefore, positive self-efficacy is decisive in promoting exercise and improving exercise persistence. Middle-aged and elderly people with high self-efficacy might be more confident in keeping exercise and bravely face the difficulties in sports participation.

Furthermore, studies have indicated that sports participation could predict several variables of living conditions, including positive life attitudes (Baldwin et al., 2013), living habits (Kostecka et al., 2017), and health promotion behaviors (Lee and Oh, 2020). Su et al. (2018) held that exercise with a resistance band could improve the health promotion lifestyle and health-related physical fitness of the elderly. Exercising for an hour and a half to 2 h daily is conducive to advancing self-actualization, dietary habits, and stress management in health promotion behaviors (Oh, 2011). Based on the research of Fu (2017), the longer the years spent on tennis participation, the higher the level of nutrition and health responsibility in health promotion behaviors, implying that the lifestyle of the elderly is healthier and more reasonable when they do exercises for more years. The study conducted by Kim (2010) on the difference between the exercise frequency of adult females in commercial fitness centers and their health promotion behaviors indicated that those who exercised more than three times a week showed a significantly higher level of health promotion behaviors on the dietary habits, exercise, personal hygiene, disease prevention, interpersonal relationships, and stress management than those who exercised less than three times a week, which is consistent with this study. The research on the effect of physical activity level on chronic diseases of some Chongqing residents carried out by Ding (2016) indicated that physical activity level has a significant positive correlation with the morbidity rate of diabetes, dyslipidemia, and metabolic syndrome.

The habit of sports participation is developed through long-term physical exercise. Middle-aged and elderly people often participate in leisure activities because they have experienced self-actualization, developed their potential, and obtained a sense of accomplishment and emotional satisfaction through physical exercise (Zheng et al., 2020). Such improved self-actualization will affect the improvement of health promotion behaviors. Therefore, sports participation is closely related to health promotion behaviors. Specifically, sports participation could positively influence the healthy living habits of middle-aged and elderly people. Given the results of previous studies and this study, the author believes that we should organize and plan some popular sports experience activities while vigorously promoting and advocating physical exercises for middle-aged and elderly people to get more of them to participate in physical exercise and develop healthier behaviors. However, other studies have pointed out that the greater the intensity of exercise and the longer the exercise time will reduce people’s happiness and bring psychological burden to exercise participants. Therefore, for middle-aged and elderly people, proper exercise can promote a healthy lifestyle (Hu, 2019).

This study has verified that self-efficacy could directly or indirectly predict health promotion behaviors with sports participation as an intermediary variable. Sports participation plays an intermediary role as an essential bridge between self-efficacy and health promotion behavior. Self-efficacy might affect the level of sports participation of middle-aged and elderly people and acts as a positive predictor of health promotion behaviors. According to the self-efficacy theory, improving self-efficacy will improve the sense of self-worth, self-confidence, and sports motivation to meet their own psychological needs to adhere to physical exercise more (Yang et al., 2020). Sports participation might improve the health promotion behaviors of middle-aged and elderly people with a high level of self-efficacy who had a positive emotional experience of physical exercise in the past and are satisfied with the pleasure they gained and more active in participating in sports activities.

As one of the health promotion behaviors, physical exercise makes the participants enjoy physical and mental happiness and helps them promote physical and mental health, build harmonious interpersonal relationships, and realize self-fulfillment, thereby comprehensively improving their health promotion behaviors through implicit and explicit behaviors. Hence, comprehensively enhancing the health promotion behaviors of middle-aged and elderly people could improve their self-efficacy and sports participation, which is also an effective way to improve their health promotion behaviors. More importantly, the impact of self-efficacy on health promotion behaviors might be complicated due to various internal and external factors. For example, the presence of chronic diseases and the awareness of health knowledge (Holden et al., 2022) and the surrounding built environment (Wang et al., 2020). Therefore, it is suggested that future studies emphasize moderating and intervening variables to lay a practical foundation for a comprehensive interpretation of the influence of health promotion behaviors of middle-aged and elderly people.

## Theoretical and practical implications

### Theoretical contribution

It enriched and extended the application fields of the theoretical model of health behavior promotion. Although self-efficacy was initially used to explain health promotion behaviors in people with chronic diseases (Keller et al., 2016), it has been gradually expanded and applied in areas such as medication adherence and health behavior control (Martinez et al., 2020). However, the impact of self-efficacy on health promotion behaviors has yet to be explored from the perspective of the level of sport participation in healthy groups. Besides, the mediating effects of sport participation have yet to be examined. The study constructs a model of factors affecting health promotion behaviors, elucidates the mechanisms by which self-efficacy influences health promotion behaviors, and provides new empirical evidence on the critical role of self-efficacy in middle-aged and elderly populations. Furthermore, it enriches the existing research literature on self-efficacy.

China is currently encountering the grave challenge of accelerating the aging process. It is urgent to promote healthy aging. Based on this reality, the study empirically verifies the mediating role of sport participation in enhancing health promotion behaviors among middle-aged and elderly adults. It also explores the impact of middle-aged and elderly adults’ sport participation on their health promotion intentions, making a theoretical contribution to improving the health status of elderly adults.

### Practical implications

It is clear from the results that self-efficacy significantly affects health promotion behaviors. Therefore, we should enhance the self-efficacy of middle-aged and elderly adults, which is mainly influenced by four factors. They are direct experience, alternative experience, verbal persuasion, and other psycho-social factors, respectively (Fan et al., 2014), for example, setting up behavioral shaping, alternative experience, motivational evaluation, social and family support, etc., to form an integrated “cognitive-motivational-emotional-behavioral” as an integrated intervention program to positively influence the self-efficacy of middle-aged and elderly people from different entry points. The government, community organizations, and elderly care institutions can help the elderly form a scientific awareness of health, change their poor lifestyles, and reduce the pressure of medical expenses through publicity and education.

This study also constructs the mediating role of middle-aged and elderly people’s sports participation in self-efficacy and health-promoting behavior. Government management organizations can continue to promote fitness for all, use sports participation as an essential means to enhance people’s healthy lifestyles, and use public places such as communities, city squares, and parks to create dedicated spaces and fixed places for sports participation. Besides, they also organize sports events to enhance residents’ sports participation enthusiasm. The act of sport should be made a part of life to enhance people’s health-promoting behaviors and promote healthy aging.

## Limitations of the study and future research directions

There are also some limitations to this study. First, due to the limitations of actual conditions, this study adopts the convenience sampling method and collects 591 samples in five cities in Henan Province. Therefore, this study lacks a sample in the age range of 45–49 years. Moreover, it only focuses on people who have physical training in parks, leisure centers, etc., who are generally healthy, with a high level of sports participation and exercise motivation. In future research, the differences in survey samples should be considered, the number of samples should be further increased, and the scope of research should also be expanded to improve the value of related research. Second, this study uses self-reported questionnaires to collect data. The limitations of a cross-sectional study make the causal relationship between variables unreliable. Therefore, a chase experiment or experimental study paradigm can be employed in future studies to verify the research results further. Finally, this study verifies the effect of self-efficacy on the health promotion behavior of middle-aged and elderly people, in which sports participation plays a mediating role. The health promotion behavior of middle-aged and elderly people is complex, and the influencing factors are diversified. For example, people’s health level (Vella et al., 2019) and health literacy (Lee and Oh, 2020) significantly influence health promotion behavior. In terms of the external environment, the surrounding environment (Yan et al., 2018) can also influence health promotion behavior. Therefore, it is suggested that future studies can further explore other variables that affect the health promotion behavior of middle-aged and elderly people.

## Conclusion

(1) As indicated by the empirical tests, self-efficacy could significantly and positively predict health promotion behaviors of middle-aged and elderly people. Improving the level of self-efficacy could advance the level of health promotion behaviors of middle-aged and elderly people.

(2) Self-efficacy could positively predict the level of sports participation; sports participation could positively predict health promotion behaviors. The SEM built in this study has verified the partial intermediary role of sports participation, i.e., self-efficacy affects health promotion behaviors in the following two ways: directly affecting health promotion behaviors and indirectly affecting them through sports participation.

## Data availability statement

The original contributions presented in this study are included in the article/supplementary material, further inquiries can be directed to the corresponding author.

## Ethics statement

The studies involving human participants were reviewed and approved by the Ethics Committee of Anyang City Third People’s Hospital in China. The patients/participants provided their written informed consent to participate in this study.

## Author contributions

NC was the leader of the research group that conducted the study and organized the database. JZ assisted in analyzing and interpreting the data and participated in the revision of the manuscript. ZW oversaw the study. All the authors read and approved the final version of the manuscript.
